# Factors affecting target caloric achievement and calorie intake improvement: the nutrition support team's role

**DOI:** 10.3389/fnut.2023.1249638

**Published:** 2024-01-05

**Authors:** Jeong Bin Bong, So-Yeong Kim, Han Uk Ryu, Hyun Goo Kang

**Affiliations:** ^1^Department of Neurology, Chosun University School of Medicine, Gwangju, Republic of Korea; ^2^Department of Preventive Medicine, College of Medicine, Chosun University, Gwangju, Republic of Korea; ^3^Department of Neurology and Biomedical Research Institute, Jeonbuk National University Medical School and Hospital, Jeonju, Republic of Korea

**Keywords:** nutritional support, malnutrition, caloric restriction, enteral nutrition, neurology

## Abstract

**Background:**

The nutrition support team (NST) works to improve malnutrition in hospitalized patients, and its role is expanding as more hospitals adopt NST. This study aimed to identify the clinical characteristics of NST-referred patients admitted to a tertiary hospital. The study focused on two groups: those who achieved the target calories, approximately 75% or more of their caloric needs relative to their body weight regardless of the period after the first NST referral, and those who improved their calorie intake 1 week after NST therapy. This study also analyzed the important factors affecting the achievement of target calorie intake and improvement in calorie intake to discover the focus of future NST therapy.

**Methods:**

This study examined 1,171 adult patients (aged ≥18 years) who were referred to the NST from all the departments within a tertiary hospital at least twice, with a minimum one-week interval between referrals, between January 1, 2019, and December 31, 2020. The study participants consisted of patients receiving <75% of their required caloric intake at the time of their first NST referral. Patients were categorized and compared according to whether they achieved their target calorie intake regardless of the period after the first NST referral and whether they improved their calorie intake 1 week after the NST therapy. We then identified factors affecting target caloric achievement and improvement in calorie intake.

**Results:**

The group that achieved the target calorie intake had a lower proportion of neuro department patients (31.3%), a higher proportion of patients receiving intensive care unit (ICU) care (31.9%), and a longer ICU stay (*p* < 0.001) than the group that did not achieve the target calorie intake. Neuro department admission negatively affected target caloric achievement [adjusted odds ratio (aOR) = 0.305, 95% confidence interval (CI) = 0.150–0.617], whereas the length of ICU stay positively affected target caloric achievement (aOR = 1.025, 95% CI = 1.007–1.043). The proportion of neuro department patients was also low (42.5%) in the group with improved calorie intake 1 week after NST therapy. Neuro department admission was a negative factor (aOR = 0.376, 95% CI = 0.264–0.537) affecting the improvement in calorie intake.

**Conclusions:**

NST therapy significantly improved clinical outcomes for inpatients at nutritional risk. Because achieving target calories and improving calorie intake in neuro department patients is difficult, it is necessary to actively refer them to NST to achieve the target calories and improve calorie intake. Furthermore, because a longer ICU stay positively affects target calorie achievement, the system for ICU nutrition therapy should be expanded and implemented for general-ward patients, including neurological patients.

## 1 Introduction

Developed countries, such as the United States, began establishing a nutrition support team (NST) in the late 1960s to improve the malnutrition issue of hospitalized patients (1). South Korea also began implementing intensive nutrition treatment in the late 1990s by creating teams of experts interested in nutrition support. As medical insurance began to cover nutrition treatment in August 2014, it became possible to run NST more systematically. Since then, many hospitals with inpatient beds get interested in organizing and operating NSTs, and the importance of NSTs is growing steadily. While the number of hospitals interested in NST therapy has increased, there is a lack of studies examining the qualitative benefits of NST therapy for the patient's nutrition and the critical factors for increasing nutritional intake (2).

Additionally, patients with neurological diseases are at higher risk of malnutrition due to factors like dysphagia, reduced consciousness, cognitive decline, muscle weakness, and neuropsychological issues (3). However, studies on specialized nutritional support methods for this patient group are currently limited. Therefore, the present study aimed to identify the future focus of NST therapy by analyzing how NST activities influence patients admitted to tertiary care institutions with an emphasis on neurological patients to achieve target calories or improve calorie intake, and critical factors affecting target caloric achievement and improvement in calorie intake.

## 2 Methods

### 2.1 Study participants

This study included 5,153 adult patients (aged ≥18 years) referred to the NST from all the departments within a tertiary hospital between January 1, 2019 and December 31, 2020. Among them, 1,490 patients met the inclusion criteria by being referred to NST at least twice, with a minimum one-week interval between referrals. The exclusion criteria for this study encompassed patients who had adequate caloric intake (≥75% of required calories) from the time of the first referral. A total of 309 patients meeting this exclusion criterion were excluded from the analysis. Additionally, 10 patients with insufficient nutritional evaluation data were also excluded, resulting in the final analysis of data from 1,171 patients. Information on referred patients was analyzed retrospectively using electronic medical records and NST patient care sheets. This study was approved by the hospital's Institutional Review Board (2022-09-001). The requirement for consent was waived due to the study's retrospective nature (2022-09-001).

### 2.2 Materials and methods

Indications for referral to NST were: (1) blood albumin level ≤ 3.0 g/dl, (2) receiving enteral nutrition (EN), (3) receiving parenteral nutrition (PN), (4) being treated in an intensive care unit (ICU), or (5) deemed to require intensive nutrition therapy based on the medical opinion of the treating physician.

This study evaluated each patient's baseline information, clinical characteristics, and clinical course using electronic medical records and NST patient care sheets. The baseline information included age, sex, body mass index (BMI), clinical department, and the primary care physician‘s NST team membership status. Clinical characteristics and courses included serum albumin levels (<3.0 g/dL), utilization of EN or PN, spontaneous feeding, nil per os status, ascites, edema, jaundice, dialysis, reduced appetite, difficulty chewing, difficulty swallowing, diarrhea, constipation, presence of pressure ulcers, the initial stage of pressure ulcers at first NST referral, change in the stage of pressure ulcers between first NST referral and discharge, provision ICU care, length of ICU care, and acute physiology and chronic health evaluation II score at ICU admission. Additionally the assessment involved daily caloric intake measured by a clinical nutritionist, achievement of target calories, number of weeks to achieve the target calories (only for patients who achieved it), improvement in calorie intake at the second NST referral 1 week after the first referral, and basic blood test results.

The daily caloric intake at the initial NST referral was not determined based on nutritional support. Subsequent nutritional support following the NST referral was administered according to the recommendations of the NST. Commercial formulas were used for both enteral and parenteral nutrition in adherence to this hospital regulations, with the specific type determined by factors such as the underlying disease (e.g., diabetes or kidney disease) and the type of intravenous line (peripheral or central). For patients engaging in spontaneous feeding, a total of 2100 kcal/day was supplied, including regular meals and provided snacks. The calorie requirement was calculated based on the individual patient's weight status, taking into account factors such as obesity or underweight.

Inpatient medical departments were categorized into the neuro (neurology and neurosurgery) and non-neuro (allergy and clinical immunology, cardiology, endocrinology, gastroenterology, hemato-oncology, infectology, nephrology, rheumatology, pulmonology, cardiothoracic surgery, general surgery, orthopedic surgery, and plastic surgery). They were also categorized into internal medicine departments (allergy and clinical immunology, cardiology, endocrinology, gastroenterology, hemato-oncology, infectology, nephrology, rheumatology, pulmonology, and neurology) and surgical departments (cardiothoracic surgery, general surgery, orthopedics, plastic surgery, and neurosurgery). When a primary care physician of patient completed NST-related training accredited by the Health Insurance Review & Assessment Service and actively participated as a member of the NST team, they were defined as members of the NST team. EN was limited to caloric supply through a feeding tube, such as a nasogastric or percutaneous endoscopic gastrostomy tube, whereas spontaneous feeding was defined as oral feeding without a tube. The presence and stage of a pressure ulcer were classified into five stages (stages 0–4) using the pressure ulcer assessment sheet in the medical records. The target calorie was defined as ≥75% of the caloric requirement based on weight-based calorie needs. Target caloric achievement was defined as attaining ≥75% of the calorie requirement regardless of the period it is achieved after the first NST referral. The calorie intake improvement was defined as any degree of enhancement in calorie provision when comparing the calorie supply at the second NST referral, referred 1 week after the first NST referral, to the initial calorie supply.

To compare patients' clinical characteristics and courses in this study, patients were divided into a “target caloric achievement group” and a “non- achievement group.” In addition, the patients were divided into a “calorie intake improvement group” and a “non- improvement group.” Then, this study compared the clinical characteristics and courses of the two groups respectively, and identified factors affecting the achievement of target calories and improvement in calorie intake.

### 2.3 Data analysis methods

First, this study observed differences in demographics, clinical characteristics, nutritional status, general conditions, and laboratory findings between target caloric achievement group and non- achievement group, and between calorie intake improvement group and non- improvement group. This study further analyzed factors that influenced the differences between the two groups, respectively. Pearson's chi-square or Fisher's exact test was used for categorical variables, whereas the *t*-test was used for continuous variables. Pearson's chi-square test was performed, and when the expected frequency of each cell was <5, >20% of the cells were interpreted as Fisher's exact test values. In addition, this study performed a multivariate logistic regression analysis to identify factors influencing the target caloric achievement group and calorie intake improvement group. Statistical significance was set at *p* < 0.05 (two-tailed). All statistical analyses were performed using SPSS (version 26.0; IBM Corp., Armonk, NY, USA).

## 3 Results

This study analyzed and compared the target caloric achievement group with the non-achievement group, among the 1,171 patients referred to NST, aiming to determine the difference between the two groups (Table 1). Of all patients, 37.2% achieved their target calories within an average of approximately 1.62 weeks. The target caloric achievement group had more female patients (44.0% vs. 35.8%), was older (70.8 ± 13.8 vs. 68.9 ± 14.7), had a lower BMI (21.54 ± 3.69 vs. 22.85 ± 3.79), and had a higher proportion of non-neuro patients (69.0% vs. 44.1%) than the non-achievement group. Furthermore, the target caloric achievement group had a higher proportion of patients receiving treatment in the ICU (31.9% vs. 25.4%) and had longer ICU stay (29.87 ± 26.79 vs. 19.99 ± 17.26) than the non-achievement group. Notably, the target caloric achievement group had more physicians with NST training than the non-achievement group (31.0% vs. 25.6%). The target caloric achievement group had a lower proportion of those receiving EN (32.3% vs. 38.8%) and a higher proportion of those with low albumin levels (48.4% vs. 32.2%), receiving PN (60.6% vs. 51.8%), and fasting (56.4% vs. 48.2%) than the non-achievement group. In addition, the target caloric achievement group had a higher proportion of patients with edema (6.7% vs. 3.7%) than the non-achievement group; however, the proportions of patients with difficulty chewing (28.9% vs. 37.1%), difficulty swallowing (23.4% vs. 29.3%), and diarrhea (0.7% vs. 2.4%) were lower in the target caloric achievement group. The plasma hemoglobin and serum albumin levels were significantly (*p* < 0.001) lower in the target caloric achievement group than in the non-achievement group.

**Table 1:** Comparison of clinical characteristics between target caloric achievement group and non-achievement group.

	**Target caloric achievement group (*n =* 436)**	**Non-achievement group (*n =* 735)**	***P-*value**
Age (mean)	70.8 ± 13.8	68.9 ± 14.7	0.027
Sex (female)	192 (44.0)	263 (35.8)	0.005
Height	161.74 ± 14.79	164.25 ± 13.83	0.003
Weight	56.76 ± 12.07	62.20 ± 13.56	< 0.001
BMI	21.54 ± 3.69	22.85 ± 3.79	< 0.001
**Department**			
Surgery	210 (48.2)	340 (46.3)	0.527
Internal medicine	226 (51.8)	395 (53.7)	
Non-neuro^*^	301 (69.0)	324 (44.1)	< 0.001
Neuro^*^	135 (31.0)	411 (55.9)	
ICU treatment	139 (31.9)	187 (25.4)	0.017
Length of ICU stay (days)	29.87 ± 26.79	19.99 ± 17.26	< 0.001
APACHE II score	14.42 ± 7.33	14.29 ± 8.13	0.890
Physicians received NST training	135 (31.0)	188 (25.6)	0.046
Low albumin^**^	211 (48.4)	237 (32.2)	< 0.001
EN	141 (32.3)	285 (38.8)	0.027
PN	264 (60.6)	381 (51.8)	0.004
Spontaneous feeding	56 (12.8)	114 (15.5)	0.211
NPO	246 (56.4)	354 (48.2)	0.006
Ascites	2 (0.5)	4 (0.5)	0.843
Edema	29 (6.7)	27 (3.7)	0.021
Jaundice	2 (0.5)	5 (0.7)	0.634
Dialysis	17 (3.9)	31 (4.2)	0.790
Bad appetite	50 (11.5)	86 (11.7)	0.904
Difficulty chewing	126 (28.9)	273 (37.1)	0.004
Difficulty swallowing	102 (23.4)	215 (29.3)	0.029
Diarrhea	3 (0.7)	18 (2.4)	0.028
Constipation	0 (0.0)	4 (0.5)	0.123
Pressure ulcer	310 (71.1)	450 (61.2)	0.001
**Pressure ulcer stages at NST referral**			
Stage 0	13 (4.0)	21 (4.5)	
Stage 1	209 (64.3)	325 (69.6)	
Stage 2	87 (26.8)	112 (24.0)	0.108
Stage 3	13 (4.0)	6 (1.3)	
Stage 4	3 (0.9)	3 (0.6)	
**Changes in pressure ulcer stages** ^***^			
Improved	10 (3.1)	15 (3.2)	
No change	284 (87.4)	413 (88.4)	0.843
Aggravated	31 (9.5)	39 (8.4)	
Average number of weeks to target caloric achievement^****^	1.62 ± 1.64	-	-
**Laboratory findings**			
WBC (10^∧^3/μ*l*)	11.23 ± 5.92	11.11 ± 4.99	0.710
Hb (g/dL)	10.15 ± 2.08	10.86 ± 2.17	< 0.001
Na (mEq/L)	139.47 ± 6.80	140.18 ± 6.55	0.077
K (mEq/L)	3.70 ± 0.66	3.71 ± 0.60	0.774
Cl (mEq/L)	104.31 ± 7.22	104.90 ± 6.82	0.163
Ca (mEq/L)	7.87 ± 0.76	8.13 ± 0.69	< 0.001
Mg (mEq/L)	2.15 ± 0.38	2.09 ± 0.33	0.060
P (mg/dL)	2.96 ± 1.19	3.01 ± 1.42	0.714
ALT (U/L)	42.77 ± 98.83	36.49 ± 94.92	0.287
AST (U/L)	60.15 ± 119.12	60.06 ± 212.67	0.994
Glucose (mg/dL)	140.29 ± 69.03	137.07 ± 68.45	0.437
Albumin (g/dL)	3.07 ± 0.53	3.27 ± 0.54	< 0.001
Cholesterol (mg/dL)	131.59 ± 44.27	140.64 ± 45.40	0.007
Triglyceride (mg/dL)	112.05 ± 89.05	113.31 ± 77.44	0.837
CRP (mg/dL)	10.07 ± 9.22	8.39 ± 8.22	0.002
BUN (mg/dL)	24.62 ± 16.27	23.52 ± 16.49	0.269
Cr (mg/dL)	0.699 ± 1.11	1.07 ± 1.14	0.272

Values are presented as the number of patients (%) or mean ± standard deviation unless otherwise indicated.

* Neuro = Neurology and Neurosurgery, Non-neuro = other departments,

** Low albumin = serum albumin ≤ 3.0 g/dL,

*** Changes in pressure ulcer stages at the first time of NST referral and discharge;

**** Applicable only when target calorie is achieved, BMI, body mass index; ICU, intensive care unit; APACHE II, acute physiology and chronic health evaluation II; NST, nutrition support team; EN, enteral nutrition; PN, parenteral nutrition; NPO, nil per os or nothing by mouth; WBC, white blood cell; Hb, hemoglobin; CRP, C-reactive protein; BUN, blood urea nitrogen; Cr, creatinine.

This study analyzed and compared the calorie intake improvement group and the non-improvement group (Table 2). The calorie intake improvement group accounted for 73.6% of all patients referred to NST and had a higher proportion of female patients (40.7% vs. 33.7%), was older (70.3 ± 14.3 vs. 67.8 ± 14.7), and had a lower BMI (22.06 ± 3.77 vs. 23.19 ± 3.78) than the non-improvement group. The calorie intake improvement had a higher proportion of internal medicine (55.3% vs. 46.6%) and non-neuro (57.5% vs. 41.7%) patients than the non-improvement group. The calorie intake improvement group had a lower portion of patients receiving PN (50.6% vs. 67.6%) and those with diarrhea (1.3% vs. 3.2%) than the non-improvement group. For patients who achieved the target calories, the calorie intake improvement group achieved it faster than the non-improvement group (1.49 ± 1.66 vs. 2.71 ± 1.03).

**Table 2:** Comparison of clinical characteristics between calorie intake improvement group and non-improvement group.

	**Calorie intake improvement group (*n =* 862)**	**Non-improvement group (*n =* 309)**	***P-*value**
Age (mean)	70.3 ± 14.3	67.8 ± 14.7	0.010
Sex (female)	351 (40.7)	104 (33.7)	0.029
Height	162.97 ± 14.33	164.26 ± 13.05	0.166
Weight	59.04 ± 13.34	63.28 ± 12.56	< 0.001
BMI	22.06 ± 3.77	23.19 ± 3.78	< 0.001
**Department**			
Surgery	385 (44.7)	165 (53.4)	0.008
Internal medicine	477 (55.3)	144 (46.6)	
Non-neuro^*^	496 (57.5)	129 (41.7)	< 0.001
Neuro^*^	366 (42.5)	180 (58.3)	
ICU treatment	250 (29.0)	76 (24.6)	0.138
Length of ICU stay (days)	24.17 ± 23.06	24.32 ± 19.94	0.960
APACHE II score	14.61 ± 7.64	13.46 ± 8.27	0.299
Physicians received NST training	235 (27.3)	88 (28.5)	0.681
Low albumin^**^	334 (38.7)	114 (36.9)	0.565
EN	322 (37.4)	104 (33.7)	0.246
PN	436 (50.6)	209 (67.6)	< 0.001
Spontaneous feeding	116 (13.5)	54 (17.5)	0.085
NPO	447 (51.9)	153 (49.5)	0.480
Ascites	4 (0.5)	2 (0.6)	0.657
Edema	40 (4.6)	16 (5.2)	0.704
Jaundice	4 (0.5)	3 (1.0)	0.389
Dialysis	38 (4.4)	10 (3.2)	0.373
Bad appetite	93 (10.8)	43 (13.9)	0.141
Difficulty chewing	297 (34.5)	102 (33.0)	0.646
Difficulty swallowing	245 (28.4)	72 (23.3)	0.082
Diarrhea	11 (1.3)	10 (3.2)	0.026
Constipation	4 (0.5)	0 (0.0)	0.578
Pressure ulcer	565 (65.5)	195 (63.1)	0.441
**Pressure ulcer stages at NST referral**			
Stage 0	27 (4.5)	7 (3.5)	
Stage 1	401 (67.5)	133 (67.2)	
Stage 2	149 (25.1)	50 (25.3)	0.667
Stage 3	14 (2.4)	5 (2.5)	
Stage 4	3 (0.5)	3 (1.5)	
**Changes in pressure ulcer stages** ^***^			
Improved	17 (2.9)	8 (4.0)	
No change	524 (88.2)	173 (87.4)	0.710
Aggravated	53 (8.9)	17 (8.6)	
Average number of weeks to target caloric achievement^****^	1.49 ± 1.66	2.71 ± 1.03	< 0.001
**Laboratory findings**			
WBC (10^*w*∧^3/μ*l*)	11.10 ± 5.56	11.28 ± 4.71	0.620
Hb (g/dL)	10.59 ± 2.20	10.61 ± 2.08	0.884
Na (mEq/L)	139.69 ± 6.45	140.55 ± 7.15	0.062
K (mEq/L)	3.73 ± 0.64	3.63 ± 0.57	0.013
Cl (mEq/L)	104.41 ± 6.73	105.43 ± 7.58	0.037
Ca (mEq/L)	8.03 ± 0.74	8.05 ± 0.70	0.781
Mg (mEq/L)	2.12 ± 0.35	2.10 ± 0.35	0.687
P (mg/dL)	3.09 ± 1.39	2.68 ± 1.02	0.005
ALT (U/L)	39.73 ± 98.26	36.32 ± 91.13	0.595
AST (U/L)	60.84 ± 170.13	58.03 ± 216.46	0.818
Glucose (mg/dL)	134.74 ± 68.30	148.09 ± 68.78	0.003
Albumin (g/dL)	3.19 ± 0.54	3.23 ± 0.57	0.293
Cholesterol (mg/dL)	135.63 ± 43.11	142.72 ± 50.49	0.055
Triglyceride (mg/dL)	106.81 ± 70.97	130.34 ± 105.02	0.003
CRP (mg/dL)	9.06 ± 8.74	8.88 ± 8.34	0.755
BUN (mg/dL)	23.83 ± 15.79	24.23 ± 18.04	0.710
Cr (mg/dL)	1.05 ± 1.17	1.01 ± 1.01	0.640

Values are presented as the number of patients (%) or mean ± standard deviation unless otherwise indicated.

* Neuro = Neurology and Neurosurgery, Non-neuro = other departments,

** Low albumin = serum albumin ≤ 3.0 g/dL,

*** Changes in pressure ulcer stages at the first time of NST referral and discharge;

**** Applicable only when target calorie is achieved, BMI, body mass index; ICU, intensive care unit; APACHE II, acute physiology and chronic health evaluation II; NST, nutrition support team; EN, enteral nutrition; PN, parenteral nutrition; NPO, nil per os or nothing by mouth; WBC, white blood cell; Hb, hemoglobin; CRP, C-reactive protein; BUN, blood urea nitrogen; Cr, creatinine.

Analyzing the factors affecting target caloric achievement (Table 3), we found that old age positively influenced achieving the target calorie intake [adjusted odds ratio (aOR) = 1.029, 95% confidence interval (CI) = 1.009–1.050] and non-neuro department patients were more likely to achieve the target calorie intake than neuro department patients (aOR = 0.305, 95% CI = 0.150–0.617). Furthermore, a longer ICU stay positively influenced achieving the target calorie intake (aOR = 1.025, 95% CI = 1.007–1.043).

**Table 3:** Factors affecting target caloric achievement.

	**Crude OR (95% CI)**	***P–*value**	**Adjusted OR (95% CI)**	***P–*value**
Age	1.010 (1.001–1.018)	0.027	1.029 (1.009–1.050)	0.005
Female	1.412 (1.109–1.799)	0.005	1.045 (0.618–1.767)	0.870
BMI	0.909 (0.878–0.940)	< 0.001	0.961 (0.879–1.051)	0.386
Neuro department	0.354 (0.275–0.454)	< 0.001	0.305 (0.150–0.617)	0.001
Length of ICU stay	1.024 (1.011–1.038)	< 0.001	1.025 (1.007–1.043)	0.007
Physicians received NST training	1.305 (1.004–1.696)	0.046	1.695 (0.840–3.417)	0.140
Low albumin	1.971 (1.545–2.513)	< 0.001	1.106 (0.563–2.175)	0.770
EN	0.755 (0.588–0.969)	0.027	3.282 (0.610–17.658)	0.166
PN	1.426 (1.121–1.814)	0.004	0.897 (0.434–1.858)	0.771
NPO	1.393 (1.098–1.768)	0.006	0.430 (0.167–1.109)	0.081
Difficulty chewing	0.688 (0.533–0.888)	0.004	0.859 (0.077–9.613)	0.902
Difficulty swallowing	0.739 (0.562–0.970)	0.029	0.166 (0.022–1.280)	0.085
Pressure ulcer	1.558 (1.208–2.010)	0.001	0.736 (0.345–1.570)	0.428
Serum albumin (g/dL)	0.487 (0.387–0.614)	< 0.001	1.146 (0.537–2.449)	0.724

Values are presented as odds ratios (95% confidence intervals). BMI, body mass index; NST, nutrition support team; ICU, intensive care unit.

Analyzing the factors affecting the improvement in calorie intake 1 week after NST referral (Table 4), we found that the lower the BMI, the more improved the calorie supply (aOR = 0.939, 95% CI = 0.905–0.975), and non-neuro department admission positively influenced calorie intake improvement (aOR = 0.376, 95% CI = 0.264–0.537). In addition, receiving PN (aOR = 0.365, 95% CI = 0.269–0.495) or the presence of diarrhea (aOR = 0.263, 95% CI = 0.103–0.677) negatively affected calorie intake improvement.

**Table 4:** Factors affecting calorie intake improvement.

	**Crude OR (95% CI)**	***P–*value**	**Adjusted OR (95% CI)**	***P–*value**
Age	1.012 (1.003–1.021)	0.010	1.006 (0.996–1.016)	0.218
Female	1.354 (1.031–1.778)	0.029	1.262 (0.946–1.683)	0.113
BMI	0.925 (0.894–0.958)	< 0.001	0.939 (0.905–0.975)	0.001
Internal medicine department	1.420 (1.094–1.843)	0.008	0.976 (0.714–1.333)	0.877
Neuro department	0.529 (0.406–0.688)	< 0.001	0.376 (0.264–0.537)	< 0.001
PN	0.490 (0.373–0.644)	< 0.001	0.365 (0.269–0.495)	< 0.001
Diarrhea	0.386 (0.162–0.919)	0.032	0.263 (0.103–0.677)	0.006
Serum albumin (g/dL)	0.881 (0.695–1.116)	0.293	1.315 (0.980–1.766)	0.068

Values are presented as odds ratios (95% confidence intervals). BMI, body mass index; PN, parenteral nutrition.

## 4 Discussion

The present study compared the group that achieved the target calorie intake with the group that did not and the group that improved calorie intake 1 week after the first NST referral with the group that did not and found many significant clinical differences. Furthermore, the multivariate analysis of factors influencing the achievement of the target calorie intake and the improvement in calorie intake 1 week after NST revealed that neuro department admission was a negative factor in both cases.

In the present study, the proportion of neuro department patients was significantly lower in the target caloric achievement group than in the non-achievement group, and in the calorie intake improvement group than in the non-improvement group. The multivariate analysis also revealed that neuro department admission was a negative factor in all cases. Neuro patients are known to be more prone to malnutrition due to dysphagia, decreased consciousness, decreased cognitive function, decreased muscle strength, and various neuropsychological disturbances. Among these, dysphagia is known as the major factor (3). Furthermore, neuro patients received EN more commonly than non-neuro patients because of dysphagia or decreased consciousness (4). EN is a nutrition supply method preferred to PN if the patient's digestive function is not compromised. However, it may be impossible to supply sufficient nutrition using EN alone because there are often restrictions on increasing the amount of feeding due to the risk of aspiration and gastrointestinal complications, such as vomiting, diarrhea, regurgitation, and abdominal distention (5). Achieving the target calories or improving calorie intake could be difficult for neuro patients because of their characteristics, particularly the high incidence of dysphagia and decreased consciousness, resulting in a high proportion of patients receiving EN. Notably, the proportion of patients receiving EN was significantly higher in the group that did not achieve the target calorie intake in the present study; however, EN was not a significant factor compared with the other variables affecting the target caloric achievement.

Caloric restriction or fasting may delay the progression of some neurologic diseases or even treat them (6, 7). Preventing or treating metabolic syndrome, a major risk factor for neurologic diseases, can prevent neurologic diseases and help delay the progression of neurodegenerative diseases such as Alzheimer's and Parkinson's diseases. Therefore, neurological patients may practice caloric restriction or fasting. However, neurological patients admitted to tertiary hospitals, such as the present study's participants, have more acute neurological conditions, such as stroke and encephalitis, than chronic neurological conditions (8). The role of fasting in acute infections, including acute central nervous system infections, has not been studied enough, suggesting that fasting could be harmful in viral infection cases (9). Therefore, caloric restriction or fasting should be avoided until there is sufficient evidence that either is beneficial in most neurologic conditions, and further studies on how to ensure adequate nutrition for patients with neurologic diseases should be conducted.

Critically ill patients treated in the ICU are more vulnerable to malnutrition because they are often in a heightened pro-inflammatory state, which can worsen their nutritional status (10). Malnutrition has been known to increase morbidity and mortality, and increased intakes of energy and protein can improve the clinical outcomes of ICU patients (11). Therefore, several nutritional therapies have been proposed to improve the nutrition intake of ICU patients, and studies have shown that these therapies reduce energy deficit and length of hospital stay (12). The present study's results showed that longer ICU stay positively influenced achieving the target calories of ICU patients. This may be because more effort is put into nutritional therapy for ICU patients because they are more likely to be malnourished, and more medical nutrition therapy, such as EN and PN, is frequently used in ICU settings (13). Additionally, more aggressive NST treatment was provided to ICU patients because NST referral indications included ICU treatment in this medical institution.

For patients in the surgery department, the impact of various invasive surgical procedures and diagnostic tests, along with the physiological and chemical responses to surgery, may negatively influence nutritional status (14). The result of this study also revealed a lower proportion of surgery department patients in the calorie intake improvement group. However, this factor did not emerge as statistically significant when compared to other variables influencing calorie intake improvement. Additionally, the surgery department in this study encompassed a group including cardiothoracic surgery, general surgery, orthopedics, plastic surgery, and neurosurgery. The specific types of surgery associated with calorie intake improvement were not identified, indicating a limitation of the present study. Further follow-up studies are suggested to explore this aspect. Serum albumin and plasma hemoglobin are commonly regarded as laboratory parameters reflecting nutritional status, and they are typically measured at lower concentrations in patients with malnutrition (15). In particular, studies evaluating the role of serum albumin as a biomarker reflecting the severity of malnutrition have identified lower serum albumin concentrations in patient groups at high risk of malnutrition. However, when patients with acute illnesses are included, the predictive value of these biomarkers significantly diminishes, indicating a stronger association with inflammatory markers rather than malnutrition (16). The result of this study revealed significantly lower levels of plasma hemoglobin and serum albumin in the target caloric achievement group. It is important to note that the laboratory results used in this study represent values at the time of the first NST referral and do not reflect values after NST support. In cases where both plasma hemoglobin and serum albumin levels are low at the initial NST referral, increased attention to nutritional status by the primary care physician and NST is anticipated, potentially explaining the lower values observed in the target caloric achievement group. Furthermore, these two factors did not emerge as statistically significant contributors to target caloric achievement in our study.

Recently, the effects of NST therapy were reported in a multicenter trial (17). In inpatients at nutritional risk, the use of nutritional support contributed to improvements in the patients‘ clinical outcomes (18). In this study, the proportion of primary care physicians with NST training was significantly higher in the target caloric achievement group; however, it was not a significant factor compared with other factors affecting the target caloric achievement. Furthermore, while less than half of the patients achieved the target calories (37.2%), a substantial 73.6% showed at least some improvement in calorie intake 1 week after NST referral. Given that NST referral is expected to have a positive impact on calorie intake improvement, even if only slightly, it becomes evident that a more proactive approach to NST referrals and encouraging physicians to undergo NST training is essential.

This study had some limitations. First, because this study could not include and analyze some clinical data, such as underlying diseases and admission diagnoses of the participants, caution should be exercised in interpreting the results because other potential factors could have influenced this study's results. Second, this study could also have selection bias because data were collected and analyzed retrospectively. Third, this study is a single-center study, which may have limitations as the results cannot be extrapolated. However, despite these limitations, this study is the first to explore the effectiveness of intensive nutrition therapy in relatively many patients referred to NST and the factors that contributed to the outcomes. We expect this study's results will profoundly impact the focus and direction of NST.

## 5 Conclusion

NST therapy significantly improved clinical outcomes for inpatients at nutritional risk. Because neuro patients experience difficulties achieving target calories and improving calorie intake, actively implementing NST referral for these patients is necessary to improve calorie supply. In addition, considering that a longer ICU stay positively influences achieving target calories, implementing and expanding the system for nutrition therapy in ICU to general-ward patients, including neurological patients, is necessary. Finally, we look forward to seeing further research exploring other effective ways, such as dysphagia screening or assessment, to improve caloric supply in neurological patients.

## Data availability statement

The original contributions presented in the study are included in the article/supplementary material, further inquiries can be directed to the corresponding authors.

## Ethics statement

The studies involving humans were approved by Chosun University Hospital (2022-09-001). The studies were conducted in accordance with the local legislation and institutional requirements. Written informed consent for participation in this study was provided by the participants' legal guardians/next of kin.

## Author contributions

JB, HR, and HK: conceptualization. JB, S-YK, HR, and HK: methodology. HR: software. HK: validation, supervision, and funding acquisition. S-YK: investigation and visualization. JB and HR: resources. JB and HK: writing—original draft preparation. HR and HK: writing—review and editing. All authors read and agreed to the published version of the manuscript.
